# Medical Examiner

**Published:** 1845-02

**Authors:** 


### MEDICAL EXAMINER.

This journal has been much enlarged, and in an improved dress is to appear monthly, in future. This makes room for longer communications, and it is the intention of the editor to devote more of its pages to original articles. The Examiner is one of our contemporaries whose time of appearing we expect with pleasure. It is spirited, but courteous, independent and frank in the expression of opinions, by which it sometimes provokes an angry retort, but its tone is elevated, and its criticisms generally just. Its reputation has been steadily growing since its establishment, and it gives promise of taking a permanent place among the medical periodicals of our country. Y.

